# Supplementary figures and images for: Protein quantification and enzyme activity estimation of Pakistani wheat landraces
Source: PLoS One. 2020 Sep 23;15(9):e0239375. doi: 10.1371/journal.pone.0239375 (PMC7511017; doi:10.1371/journal.pone.0239375)

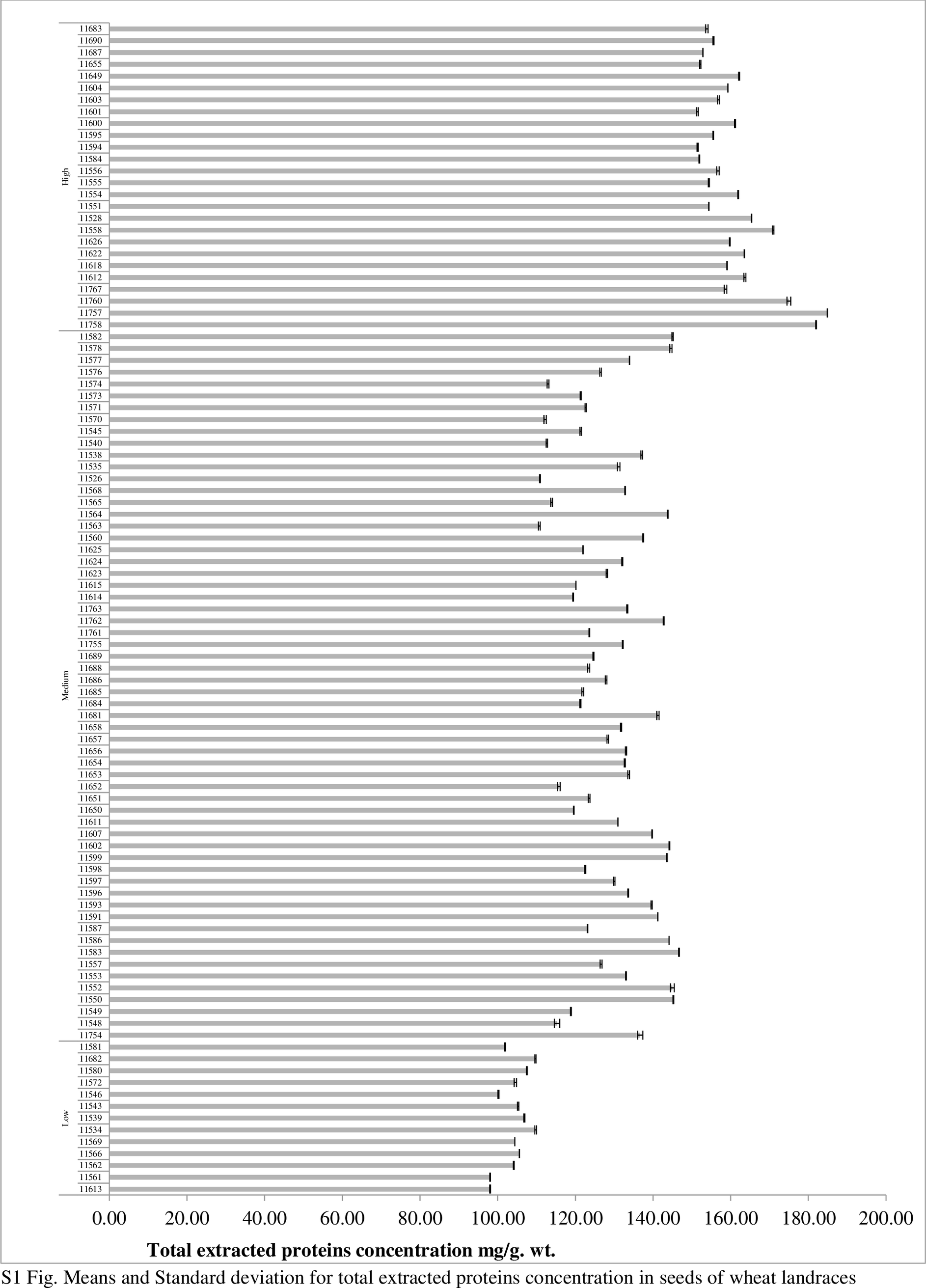

Supplement: S1 Fig — (TIFF) [file pone.0239375.s001.tiff]

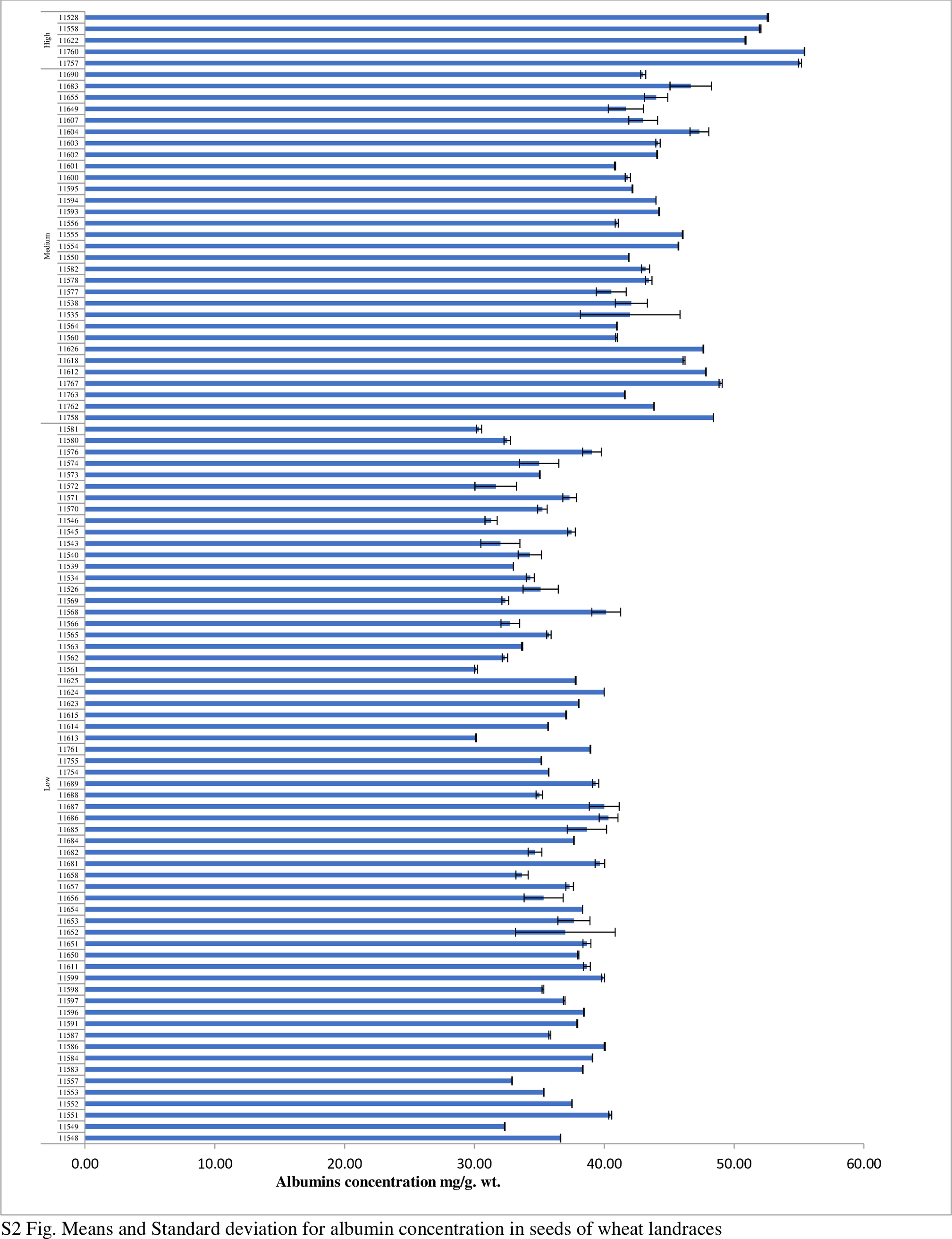

Supplement: S2 Fig — (TIFF) [file pone.0239375.s002.tiff]

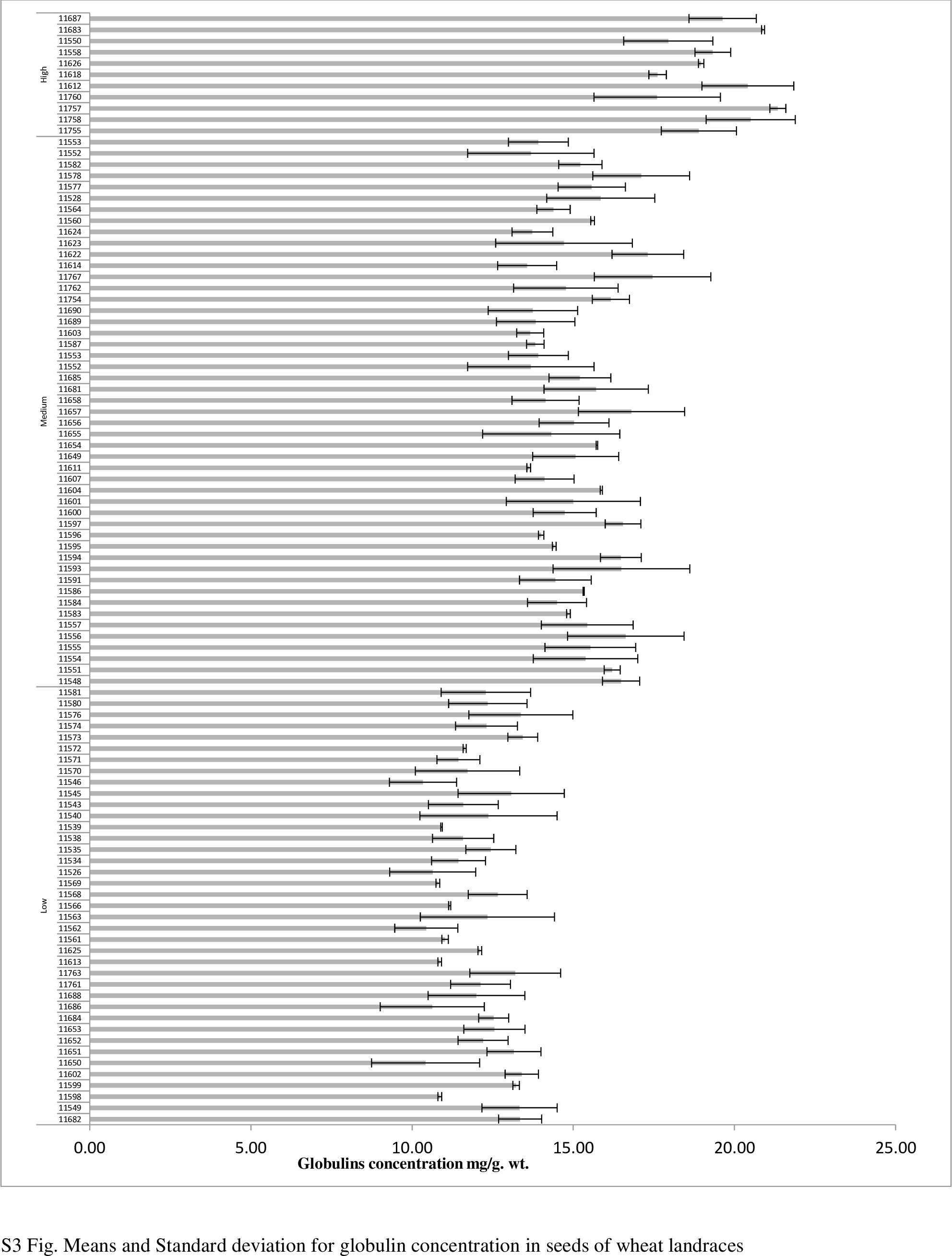

Supplement: S3 Fig — (TIFF) [file pone.0239375.s003.tiff]

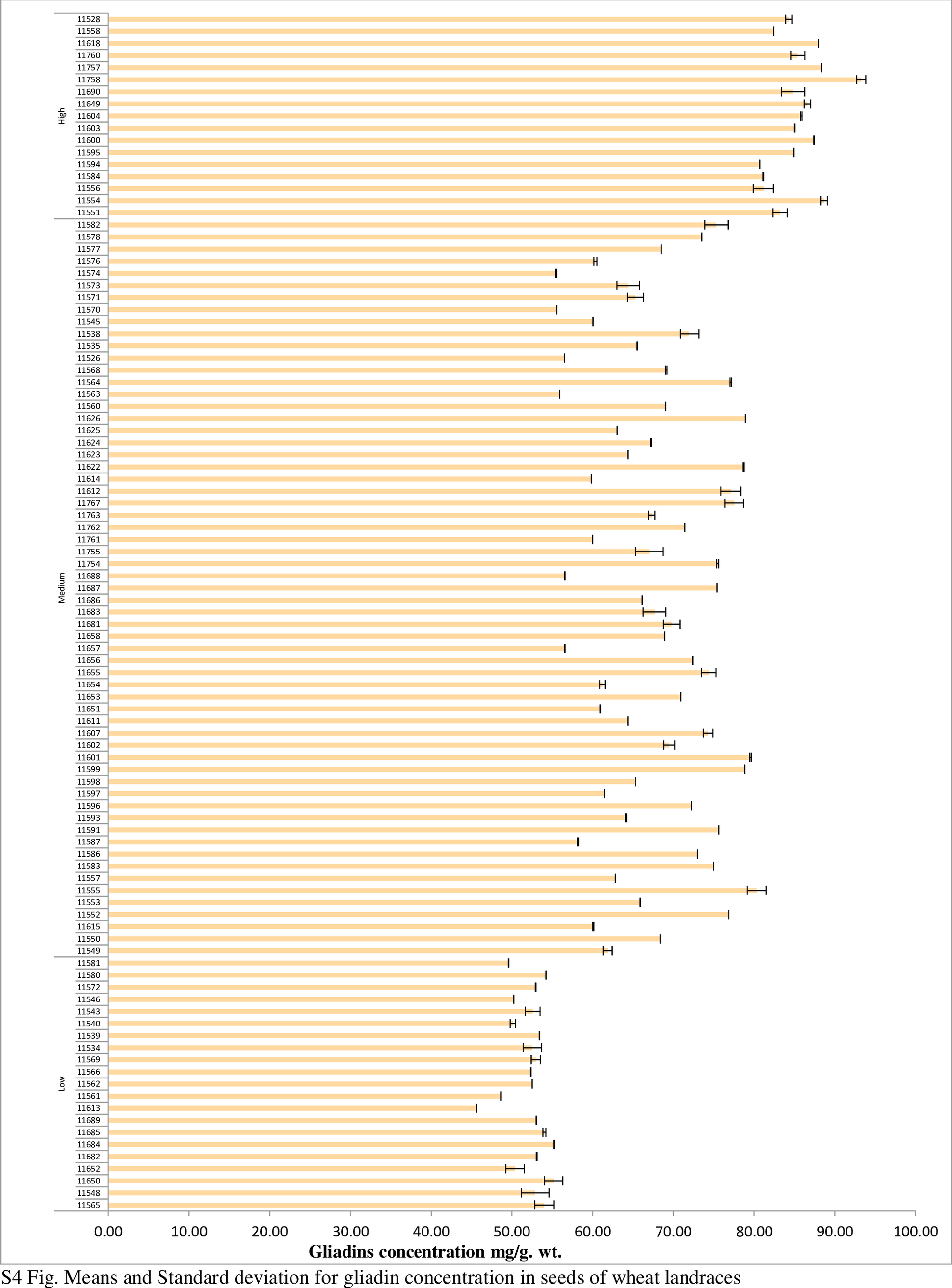

Supplement: S4 Fig — (TIFF) [file pone.0239375.s004.tiff]

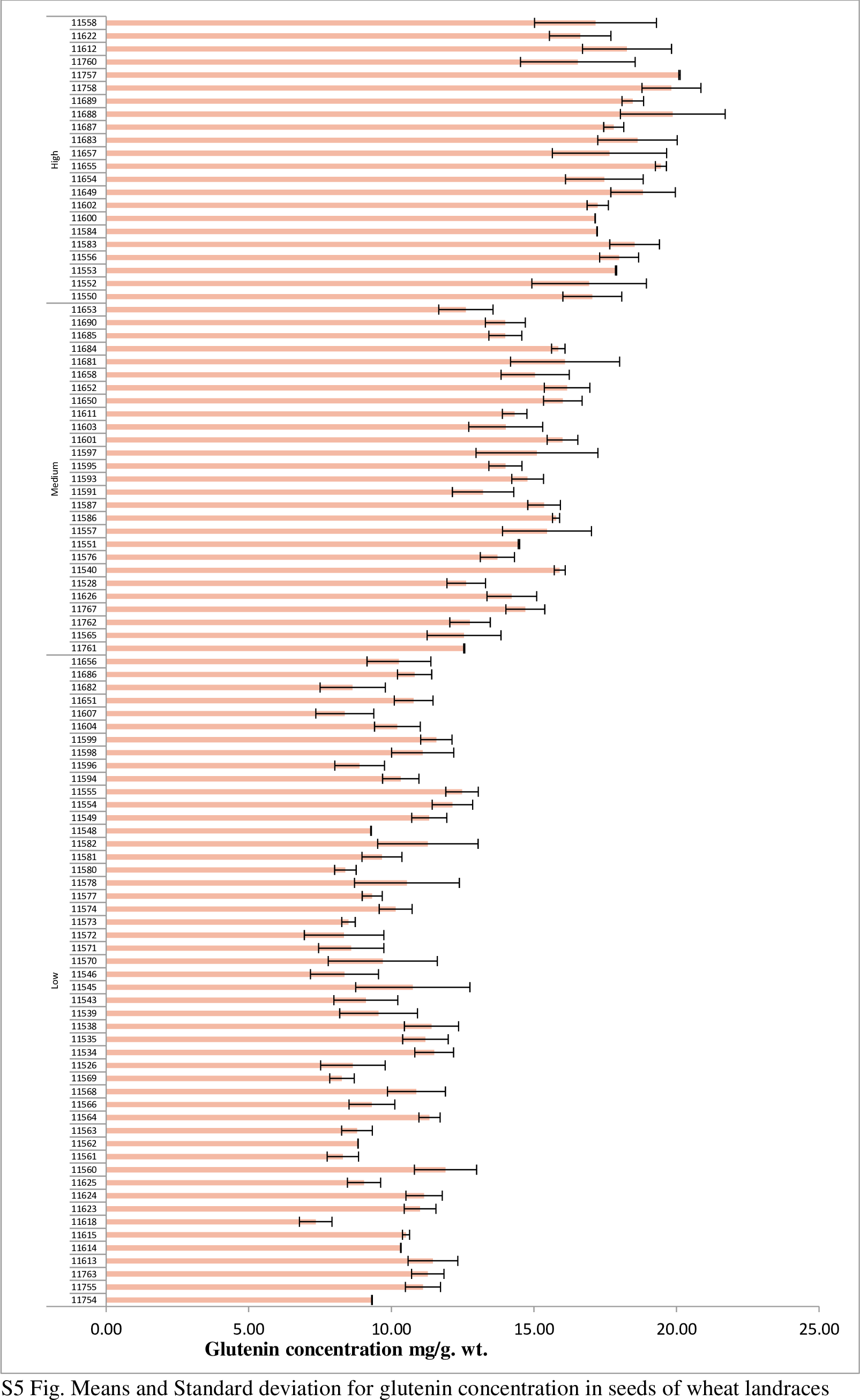

Supplement: S5 Fig — (TIFF) [file pone.0239375.s005.tiff]

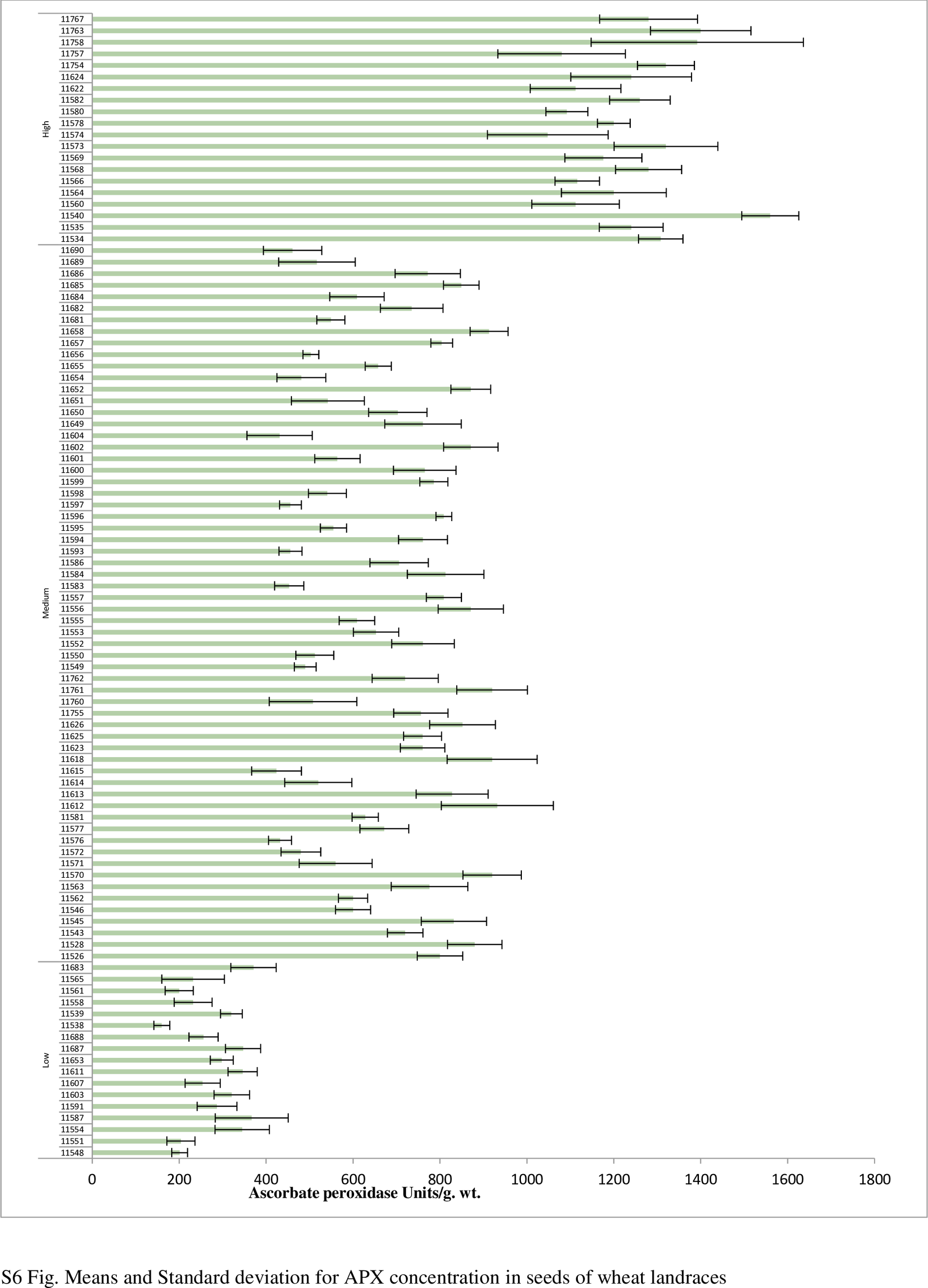

Supplement: S6 Fig — (TIFF) [file pone.0239375.s006.tiff]

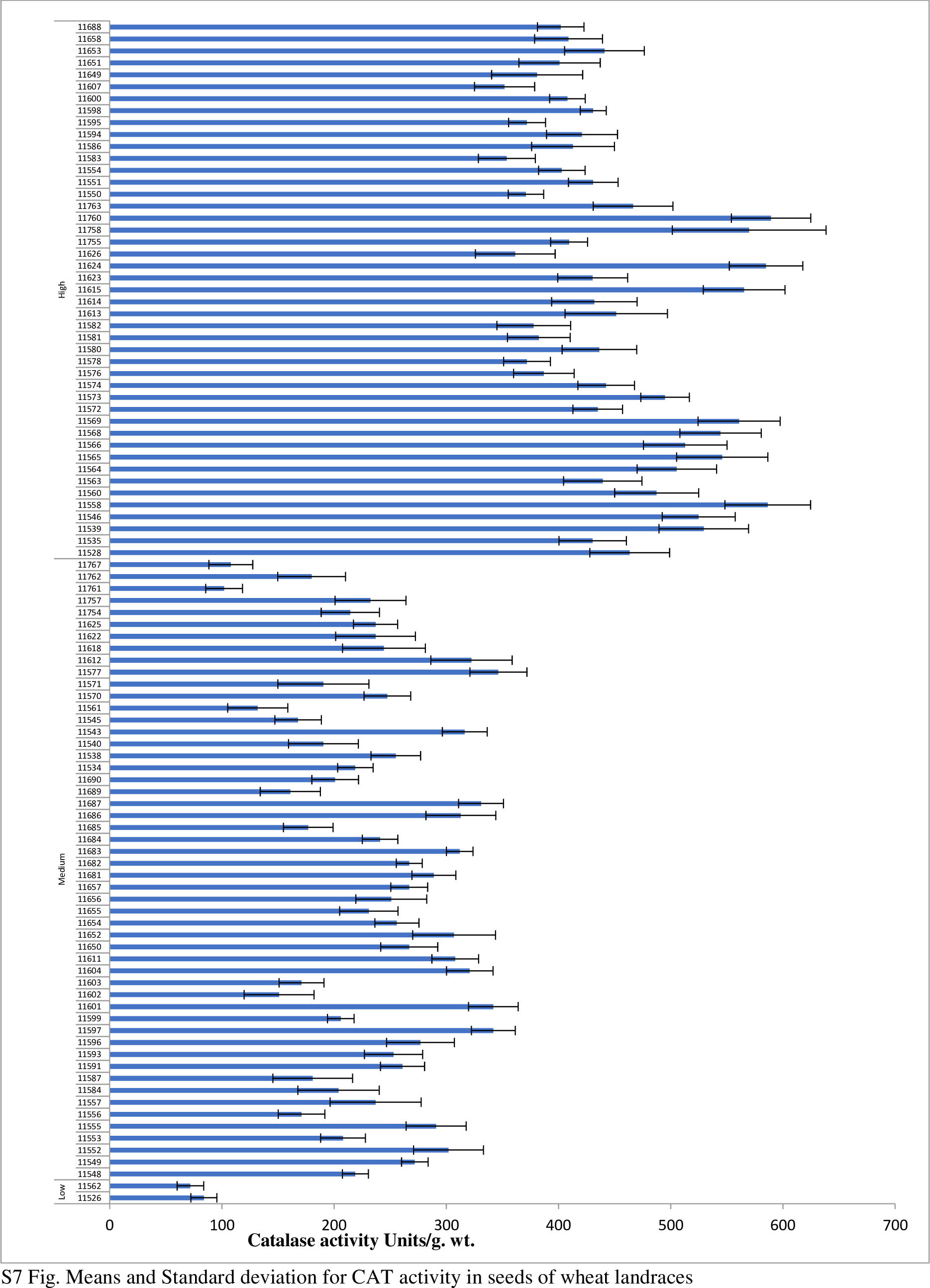

Supplement: S7 Fig — (TIFF) [file pone.0239375.s007.tiff]

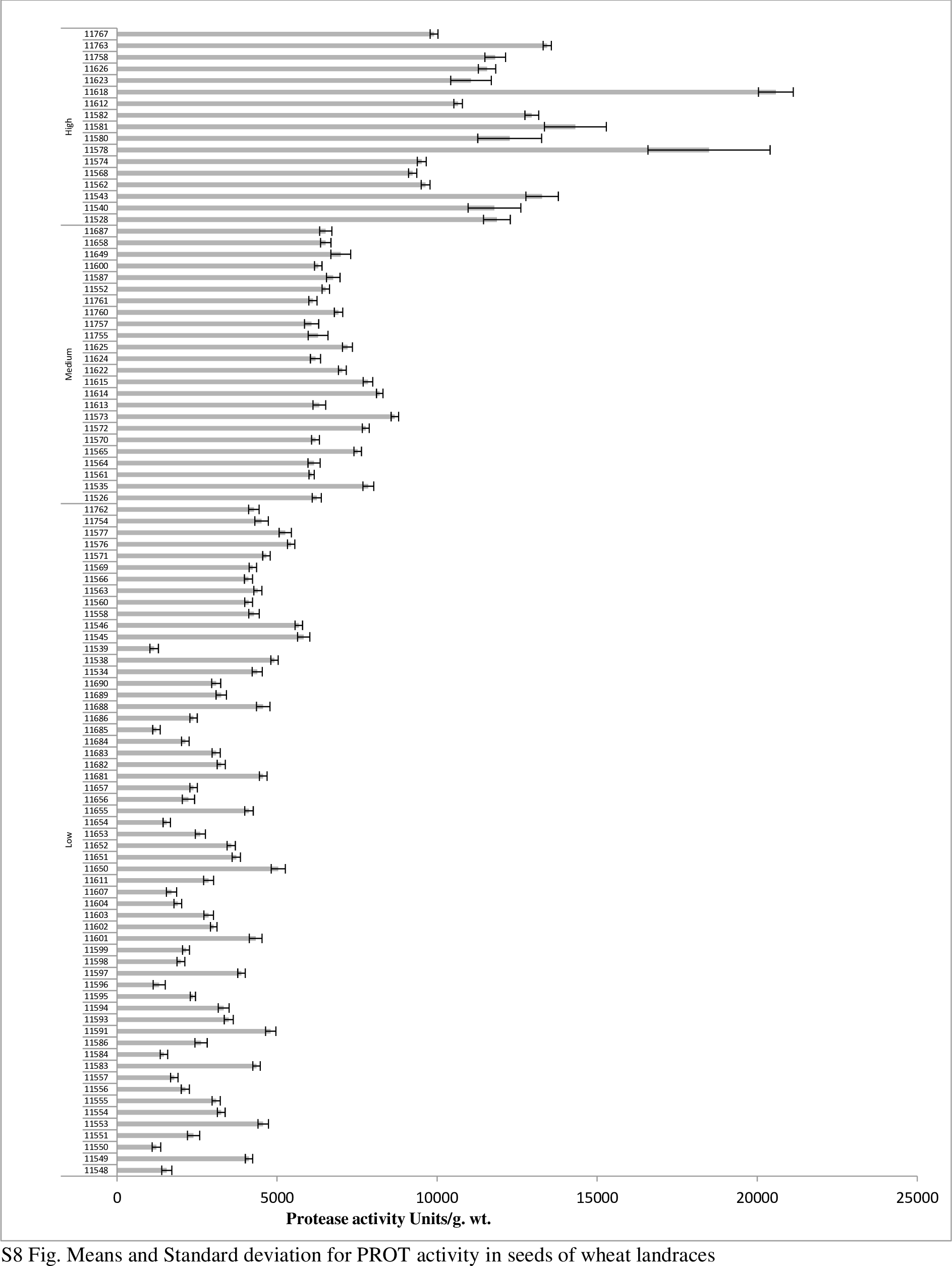

Supplement: S8 Fig — (TIFF) [file pone.0239375.s008.tiff]

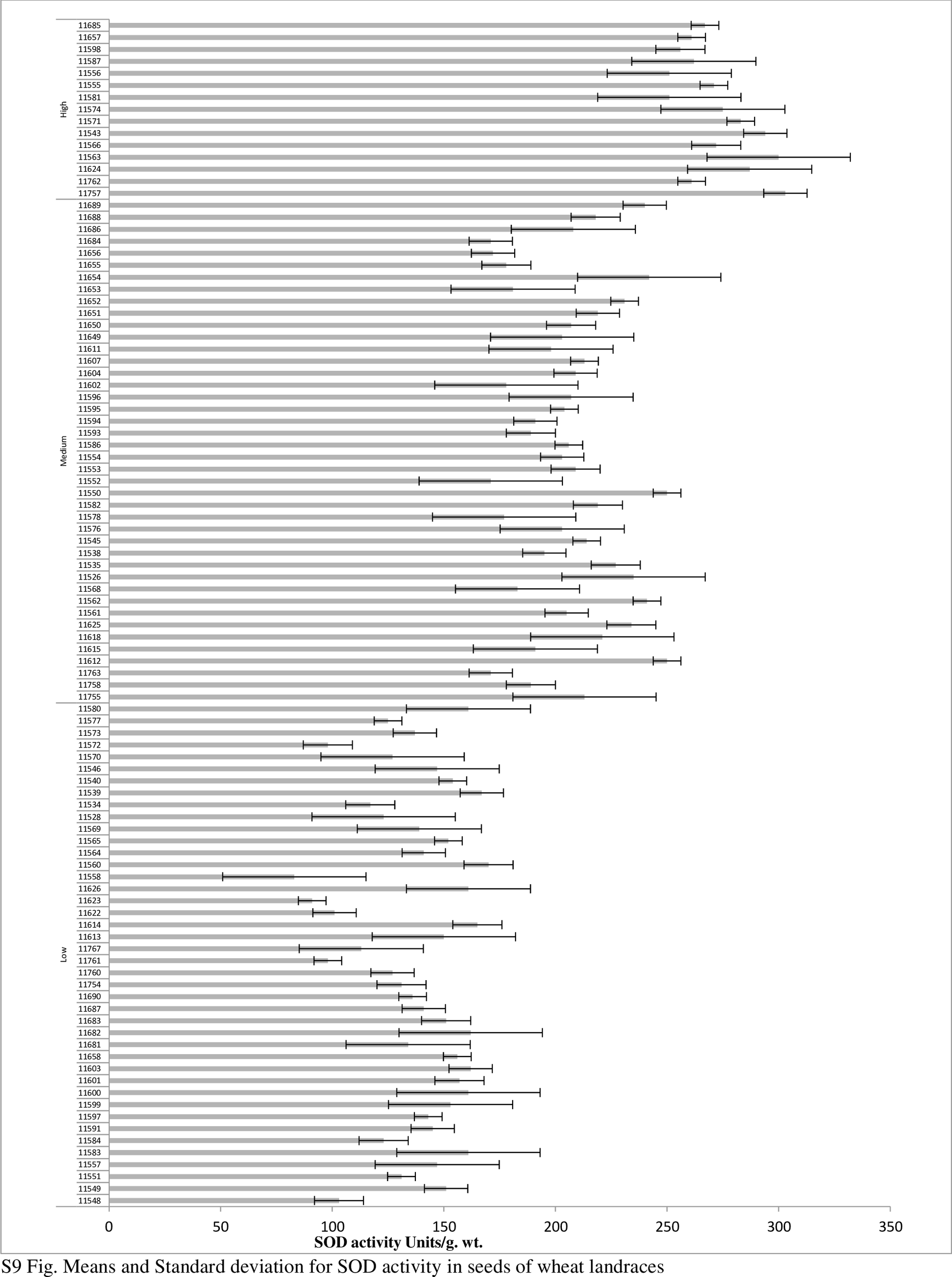

Supplement: S9 Fig — (TIFF) [file pone.0239375.s009.tiff]

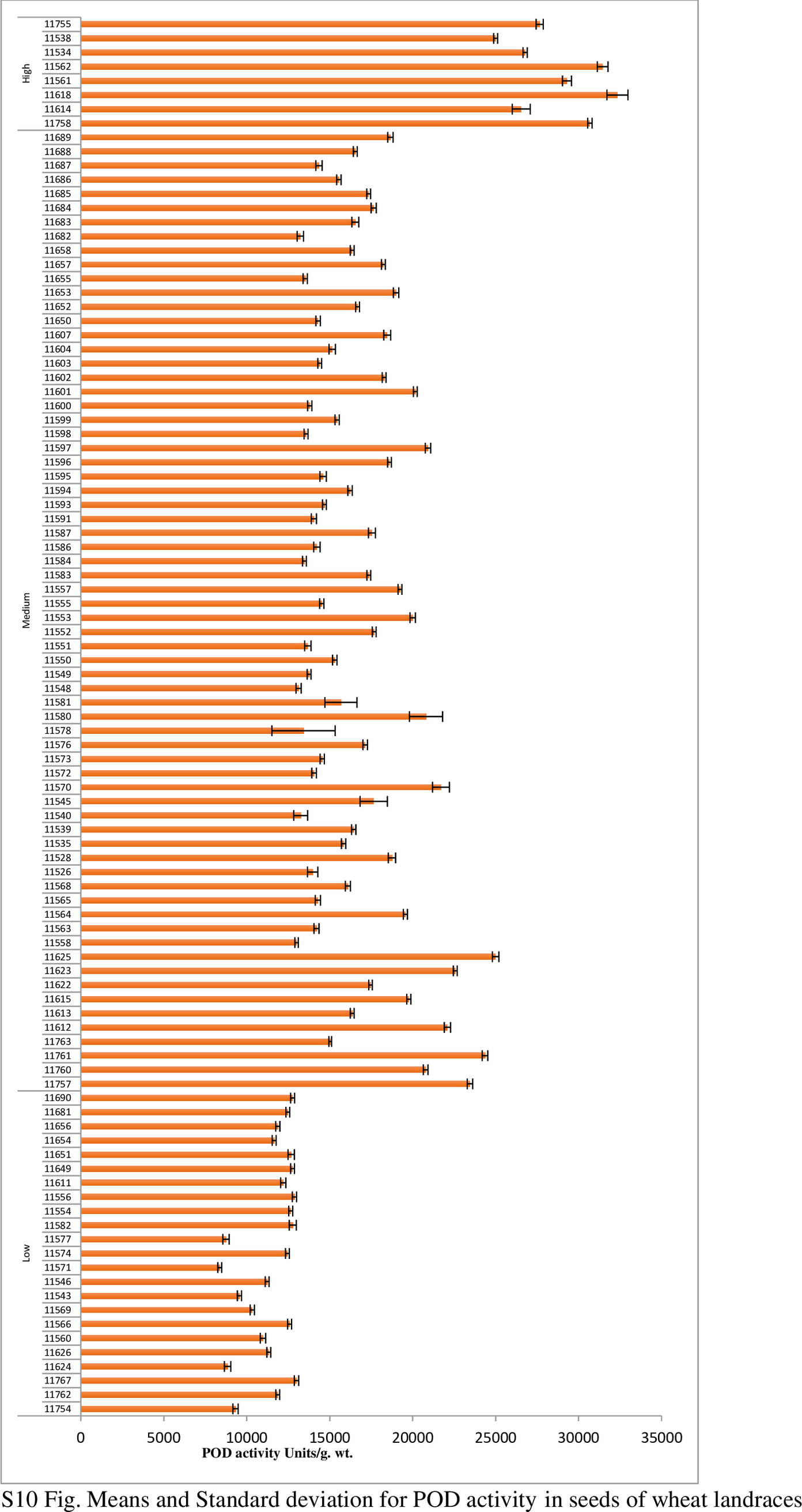

Supplement: S10 Fig — (TIFF) [file pone.0239375.s010.tiff]

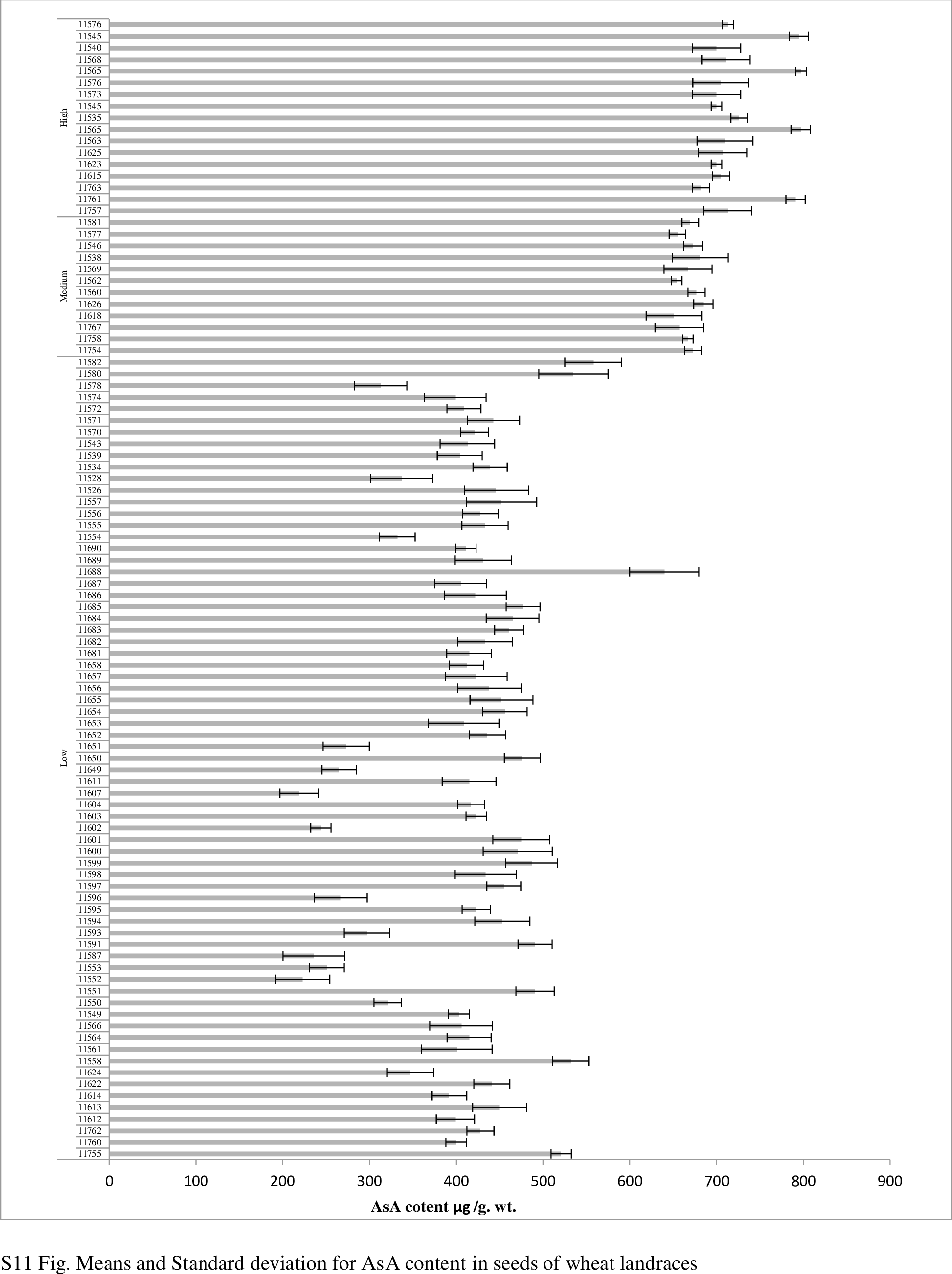

Supplement: S11 Fig — (TIFF) [file pone.0239375.s011.tiff]
