# Supplementary material for: Protein quantification and enzyme activity estimation of Pakistani wheat landraces
Source: PLoS One. 2020 Sep 23;15(9):e0239375. doi: 10.1371/journal.pone.0239375 (PMC7511017; doi:10.1371/journal.pone.0239375)
Supplement: S1 Table — (DOCX) [file pone.0239375.s012.docx]

**S1 Table :** Wheat landraces used in this study

| S. No. | Accession No. | Origin | Province | District |
| --- | --- | --- | --- | --- |
| 1.  2.  4.  3.  5.  6.  7.  8.  9.  10.  11.  12.  13.  14.  15.  16.  17.  18.  19.  20.  21.  22.  23.  24.  25.  26.  27.  28.  29.  30.  31.  32.  33.  34.  35.  36.  37.  38.  39.  40.  41.  42.  43.  44.  45.  46.  47.  48.  49.  50.  51.  52.  53.  54.  55.  56.  57.  58.  59.  60.  61.  62.  63.  64.  65.  66.  67.  68.  69.  70.  71.  72.  73.  74.  75.  76.  77.  78.  79.  80.  81.  82.  83.  84.  85.  86.  87.  88.  89.  90.  91.  92.  93.  94.  95.  96.  97.  98.  99. | 11526  11528  11534  11535  11538  11539  11540  11543  11545  11546  11548  11549  11550  11551  11552  11553  11554  11555  11556  11557  11558  11560  11561  11562  11563  11564  11565  11566  11568  11569  11570  11571  11572  11573  11574  11576  11577  11578  11580  11581  11582  11583  11584  11586  11587  11591  11593  11594  11595  11596  11597  11598  11599  11600  11601  11602  11603  11604  11607  11611  11612  11613  11614  11615  11618  11622  11623  11624  11625  11626  11649  11650  11651  11652  11653  11654  11655  11656  11657  11658  11681  11682  11683  11684  11685  11686  11687  11688  11689  11690  11754  11755  11757  11758  11760  11761  11762  11763  11767 | Pakistan  Pakistan  Pakistan  Pakistan  Pakistan  Pakistan  Pakistan  Pakistan  Pakistan  Pakistan  Pakistan  Pakistan  Pakistan  Pakistan  Pakistan  Pakistan  Pakistan  Pakistan  Pakistan  Pakistan  Pakistan  Pakistan  Pakistan  Pakistan  Pakistan  Pakistan  Pakistan  Pakistan  Pakistan  Pakistan  Pakistan  Pakistan  Pakistan  Pakistan  Pakistan  Pakistan  Pakistan  Pakistan  Pakistan  Pakistan  Pakistan  Pakistan  Pakistan  Pakistan  Pakistan  Pakistan  Pakistan  Pakistan  Pakistan  Pakistan  Pakistan  Pakistan  Pakistan  Pakistan  Pakistan  Pakistan  Pakistan  Pakistan  Pakistan  Pakistan  Pakistan  Pakistan  Pakistan  Pakistan  Pakistan  Syria  Syria  Pakistan  Pakistan  Pakistan  Pakistan  Pakistan  Pakistan  Pakistan  Pakistan  Pakistan  Pakistan  Pakistan  Pakistan  Pakistan  Pakistan  Pakistan  Pakistan  Pakistan  Pakistan  Pakistan  Pakistan  Pakistan  Pakistan  Pakistan  Pakistan  Pakistan  Pakistan  Pakistan  Pakistan  Pakistan  Pakistan  Pakistan  Pakistan | Sindh  Balochistan  Balochistan  Balochistan  Balochistan  Balochistan  Balochistan  Balochistan  Balochistan  Balochistan  Balochistan  Balochistan  Balochistan  Balochistan  Balochistan  Balochistan  Balochistan  Balochistan  Balochistan  Balochistan  Balochistan  Balochistan  Gilgit-Baltistan  Gilgit-Baltistan  Gilgit-Baltistan  Gilgit-Baltistan  Gilgit-Baltistan  Gilgit-Baltistan  Gilgit-Baltistan  Gilgit-Baltistan  Gilgit-Baltistan  Gilgit-Baltistan  Gilgit-Baltistan  Gilgit-Baltistan  Gilgit-Baltistan  Gilgit-Baltistan  Gilgit-Baltistan  Gilgit-Baltistan  Gilgit-Baltistan  Gilgit-Baltistan  Gilgit-Baltistan  Gilgit-Baltistan  Gilgit-Baltistan  Gilgit-Baltistan  Gilgit-Baltistan  Gilgit-Baltistan  Gilgit-Baltistan  Gilgit-Baltistan  Gilgit-Baltistan  Gilgit-Baltistan  Gilgit-Baltistan  Gilgit-Baltistan  Gilgit-Baltistan  Gilgit-Baltistan  Gilgit-Baltistan  Gilgit-Baltistan  Gilgit-Baltistan  Gilgit-Baltistan  Gilgit-Baltistan  KPK  KPK  KPK  KPK  Gilgit-Baltistan  Gilgit-Baltistan  --  --  Punjab  Punjab  Punjab  Punjab  Punjab  Punjab  Punjab  Punjab  Punjab  Punjab  Punjab  Punjab  Punjab  Punjab  Punjab  Punjab  Punjab  Punjab  Punjab  Punjab  Punjab  Punjab  Punjab  Balochistan  Balochistan  Balochistan  Balochistan  Balochistan  Balochistan  Balochistan  Gilgit-Baltistan  Gilgit-Baltistan | Thatta  Quetta  Quetta  Quetta  Quetta  Quetta  Quetta  Quetta  Quetta  Pishin  Pinhin  Pinhin  Dhadar  Dhadar  Mastung  Mastung  Mastung  Kharan  Mastung  Mastung  Kalat  Qila-Saifullah  Gilgit  Gilgit  Gilgit  Gilgit  Gilgit  Gilgit  Gilgit  Gilgit  Gilgit  Gilgit  Gilgit  Gilgit  Gilgit  Gilgit  Baltistan  Baltistan  Baltistan  Baltistan  Baltistan  Baltistan  Baltistan  Baltistan  Baltistan  Gilgit  Gilgit  Gilgit  Gilgit  Gilgit  Gilgit  Gilgit  Gilgit  Gilgit  Gilgit  Gilgit  Gilgit  Gilgit  Gilgit  Swat  Swat  Swat  Dir  Skardu  Gilgit  --  --  Faisalabad  Faisalabad  Faisalabad  Faisalabad  Faisalabad  Faisalabad  Faisalabad  Faisalabad  Faisalabad  Faisalabad  Faisalabad  Faisalabad  Faisalabad  Faisalabad  Faisalabad  Faisalabad  Faisalabad  Faisalabad  Faisalabad  Faisalabad  Faisalabad  Faisalabad  Faisalabad  Kharan  Kharan  Panjgur  Kech  Kech  Khuzdar  Khuzdar  Gilgit  Skardu |
