# Supplementary material for: Protein quantification and enzyme activity estimation of Pakistani wheat landraces
Source: PLoS One. 2020 Sep 23;15(9):e0239375. doi: 10.1371/journal.pone.0239375 (PMC7511017; doi:10.1371/journal.pone.0239375)
Supplement: S2 Table — (DOCX) [file pone.0239375.s013.docx]

S2 Table. Principal component analysis for selected parameters in wheat landraces

|  | F1 | F2 | F3 | F4 | F5 | F6 | F7 | F8 | F9 | F10 |
| --- | --- | --- | --- | --- | --- | --- | --- | --- | --- | --- |
| TEP | 0.513 | -0.007 | 0.024 | 0.019 | 0.037 | 0.004 | -0.124 | -0.062 | -0.145 | -0.116 |
| Alb | 0.469 | 0.067 | 0.002 | 0.036 | 0.065 | 0.033 | -0.219 | -0.010 | -0.063 | 0.816 |
| Glob | 0.438 | 0.025 | -0.042 | -0.069 | 0.070 | -0.020 | 0.201 | 0.210 | 0.820 | -0.165 |
| Gli | 0.462 | 0.020 | 0.105 | -0.029 | 0.068 | -0.013 | -0.375 | -0.178 | -0.282 | -0.530 |
| Glu | 0.323 | -0.232 | -0.190 | 0.198 | -0.169 | 0.032 | 0.748 | 0.108 | -0.380 | -0.030 |
| APX | 0.012 | 0.579 | -0.037 | 0.264 | 0.063 | -0.492 | 0.238 | -0.536 | 0.060 | 0.010 |
| CAT | 0.011 | 0.256 | 0.664 | -0.097 | 0.160 | 0.573 | 0.304 | -0.190 | -0.010 | 0.017 |
| SOD | -0.076 | -0.042 | -0.365 | 0.467 | 0.725 | 0.333 | -0.038 | -0.028 | 0.015 | -0.053 |
| POD | 0.026 | 0.201 | -0.598 | -0.521 | -0.151 | 0.410 | 0.045 | -0.370 | 0.011 | -0.003 |
| PROT | 0.012 | 0.642 | -0.100 | -0.201 | 0.171 | -0.056 | 0.036 | 0.660 | -0.254 | -0.064 |
| AsA | 0.004 | 0.292 | -0.084 | 0.587 | -0.591 | 0.381 | -0.204 | 0.111 | 0.102 | -0.066 |
| Eigenvalue | 3.714 | 1.704 | 1.193 | 1.071 | 0.945 | 0.767 | 0.635 | 0.436 | 0.331 | 0.206 |
| Variability (%) | 33.765 | 15.488 | 10.844 | 9.732 | 8.591 | 6.973 | 5.770 | 3.964 | 3.005 | 1.869 |
| Cumulative % | 33.765 | 49.253 | 60.097 | 69.829 | 78.419 | 85.392 | 91.162 | 95.126 | 98.131 | 100.000 |

**TEP= Total extracted protein, ALB= albumin; GLOB= globulin, Gli= gliadin, Glu= glutenin, APX= ascorbate peroxidase, CAT= catalase, SOD= superoxide dismutase, POD= peroxidase, PROT= protease, AsA= ascorbic acid**
