# Supplementary material for: Protein quantification and enzyme activity estimation of Pakistani wheat landraces
Source: PLoS One. 2020 Sep 23;15(9):e0239375. doi: 10.1371/journal.pone.0239375 (PMC7511017; doi:10.1371/journal.pone.0239375)
Supplement: Attachment — Submitted filename: answer to editor comments.docx [file pone.0239375.s014.docx]

Subject: Answer to reviewer comments.

Respected Editor

Thank you so much for your valuable suggestions. We have tried out best to incorporate your suggestions.

Additional Editor Comments (if provided):

Dear Authors thank you for addressing all the comments from reviewers. However, there are further suggestions to be addressed:

There are many grammatical and typographic errors in manuscript, which should be thoroughly corrected. We would recommend that you have your manuscript copy-edited by either a native-English speaking colleague or a professional copy-editing service. While you may approach any qualified individual or any professional scientific editing service of your choice, PLOS has partnered with American Journal Experts (AJE) to provide discounted services to PLOS authors. AJE has extensive experience helping authors meet PLOSguidelines and can provide language editing, translation, manuscript formatting, and figure formatting to ensure your manuscript meets our submission guidelines. If there are still language issues in text that AJE has edited, AJE will re-edit the text for free. To take advantage of this special partnership, visit the AJE website and enter referral code PLOS15 on the registration page for a 15% discount off AJE services (http://www.aje.com/c/plos15). If you are already registered with AJE, please log in and enter PLOS15 at the bottom of your researcher dashboard under ‘Join a Group.’ Please note that PLOS ONE does not receive any compensation in relation to services completed by AJE and that having the manuscript copyedited by AJE or any other editing services does not guarantee selection for peer review.

Ans: The manuscript is thoroughly read by me and Dr. Waseem Haider PhD from University of **Illinois** at **Urbana**–**Champaign.**

Authors also need to provide compelling argument to support that existing wheat varieties/advanced breeding lines in Pakistan are low or high in desired different protein types, enzymes and antioxidants. Either provide comparative data from your own studies which suggests you must have used wheat varieties/advanced breeding lines for comparison or support from published literature. For example references which you have cited on page 13- reference 31 doesn't provide needed information, however, reference 32's findings are close to what has been demonstrated in this manuscript. Though, this manuscript has added information on different types of proteins.
Authors should rewrite some of the discussion section by adding more meaningful information to bring the flow of information and related literature. It should not read more like repeat of results. Systematic highlight of how each of the protein/ antioxidant is important from either plant's or human health's perspective. This information is provided
as general overview but not for all sections. The discussion should charily outline how superiority of studied lines is in agreement or disagreement of published research, or how these findings are adding value to existing knowledge as reference 32 is one similar publication.
Page 13, where discussion starts in last paragraph- In the current study....., this whole paragraph is little confusing and requires thorough revision.

Ans: Sir, thank you so much, we have thoroughly revised the discussion. Sir, there are very few studies on screening cultivars for quality parameters. Th main focus of breeders is on yield and disease resistance parameters. We designed this study to explore the landraces deposit in gene bank for quality parameters. This study could be helpful for breeders for the selection of landraces.
