# Supplementary material for: Protein quantification and enzyme activity estimation of Pakistani wheat landraces
Source: PLoS One. 2020 Sep 23;15(9):e0239375. doi: 10.1371/journal.pone.0239375 (PMC7511017; doi:10.1371/journal.pone.0239375)
Supplement: Attachment — Submitted filename: answer to editor comments.docx [file pone.0239375.s015.docx]

Subject: Answer to reviewer comments.

Respected Editor

Thank you so much for your giving us another chance of improving our article. Sir we have tried our best to improve grammatical mistakes as well as English language. Hoping for your kind consideration. Thanks
